# Detection of three-rooted mandibular first molars on panoramic radiographs using deep learning

**DOI:** 10.1038/s41598-024-82378-8

**Published:** 2024-12-05

**Authors:** Long Jin, Ying Tang, Wenyuan Zhou, Bingbing Bai, Zezheng Yu, Panpan Zhang, Yongchun Gu

**Affiliations:** 1 Department of Radiology, Ninth People’s Hospital of Suzhou, Soochow University, Suzhou, China; 2 Department of Pathology, Ninth People’s Hospital of Suzhou, Soochow University, Suzhou, China; 3 Department of Dentistry and Central Lab, Ninth People’s Hospital of Suzhou, Soochow University, Suzhou, China; 4 The Affiliated Stomatology Hospital of Suzhou Vocational Health College, Suzhou, China

**Keywords:** Deep learning, Convolutional neural network (CNN), Panoramic radiography, Cone beam computed tomography (CBCT), Three-rooted mandibular first molar, Imaging, Oral anatomy

## Abstract

This study aimed to develop a deep learning system for the detection of three-rooted mandibular first molars (MFMs) on panoramic radiographs and to assess its diagnostic performance. Panoramic radiographs, together with cone beam computed tomographic (CBCT) images of the same subjects, were retrospectively collected from 730 patients, encompassing a total of 1444 MFMs (367 teeth were three-rooted and the remaining 1077 teeth were two-rooted). Five convolutional neural network (CNN) models (ResNet-101 and − 50, DenseNet-201, MobileNet-v3 and Inception-v3) were employed to classify three- and two-rooted MFMs on the panoramic radiographs. The diagnostic performance of each model was evaluated using standard metrics, including accuracy, sensitivity, specificity, precision, negative predictive value, and F1 score. Receiver operating characteristic (ROC) curve analyses were performed, with the CBCT examination taken as the gold standard.

Among the five CNN models evaluated, ResNet-101 demonstrated superior diagnostic performance, and the AUC value attained was 0.907, significantly higher than that of all other models (all *P* < 0.01). The accuracy, sensitivity, and specificity were 87.5%, 83.6%, and 88.9%, respectively. DenseNet-201, however, showed the lowest diagnostic performance among the five models (all *P* < 0.01), with an AUC value of 0.701 and an accuracy of 73.2%. Overall, the performance of the CNNs diminished when using image patches containing only the distal half of MFMs, with AUC values ranging between 0.680 and 0.800. In contrast, the diagnostic performance of the two clinicians was poorer, with AUC values of only 0.680 and 0.632, respectively. In conclusion, the CNN-based deep learning system exhibited a high level of accuracy in the detection of three-rooted MFMs on panoramic radiographs.

## Introduction

The permanent mandibular first molar (MFM) typically has two roots, one mesial and one distal. However, in some cases, an additional distolingual (DL) root may be present^1,2^. This root variation is influenced by ethnic background. In Mongoloid populations, the prevalence ranges from 5 to 40%, whereas in Black or White populations, the frequency is generally less than 5%^1–5^. The presence of a DL root may pose an endodontic challenge. In conventional periapical or panoramic radiographs, the overlap of the images of the two distal roots can lead clinicians to misdiagnose this root variation^4–6^. Additionally, the DL root often varies in size and is severely curved in the buccolingual plane^3,4^. These characteristics increase the risk of procedural errors or instrument separation during root canal preparation^4^. Therefore, the accurate identification of the DL root in the MFM is paramount for ensuring optimal endodontic outcomes.

In recent decades, dental cone-beam computed tomography (CBCT) has been widely used to evaluate patients’ root and canal anatomy due to its high resolution and three-dimensional nature^4–7^. It facilitates more accurate detection of the DL root and provides valuable anatomical information from multiple perspectives. However, despite the reduced scan time and radiation dose compared to conventional medical CT scans, CBCT scanners still emit higher radiation doses than a single traditional dental radiograph. Therefore, the principles of ALARA (as low as reasonably achievable) and ALADA (as low as diagnostically acceptable) should be adhered to, and the routine use of CBCT is not recommended during dental treatments^5,8^.

**Fig. 1:**
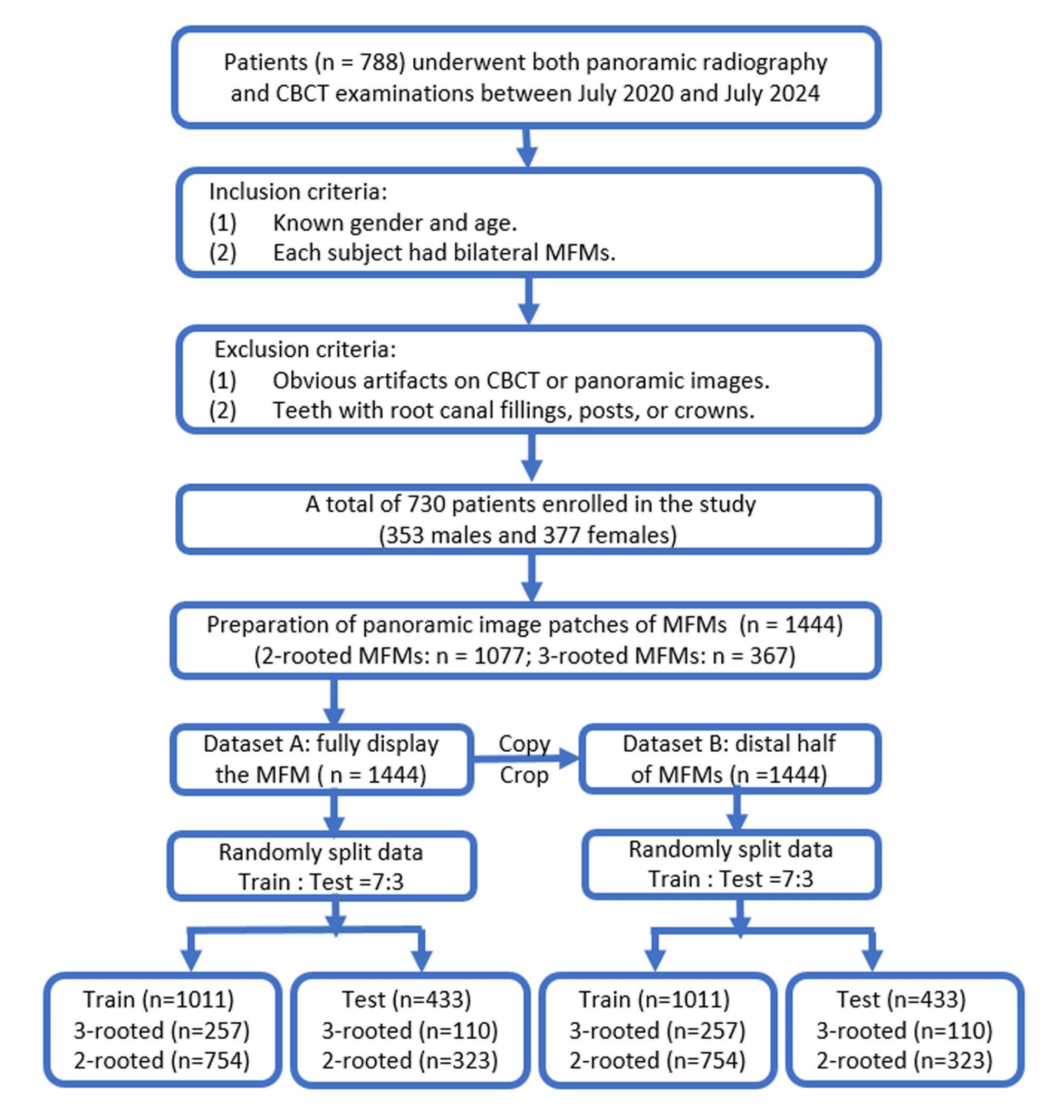
Flowchart for patient recruitment and data selection (MFM is mandibular first molar)

Recently, deep learning, a method within artificial intelligence (AI), has been increasingly applied to various aspects of dentistry, leveraging its capabilities in image analysis, pattern recognition, and data processing^9^. Deep learning models, such as convolutional neural networks (CNNs), have been utilized to analyze medical images and support diagnostic procedures due to their efficacy in handling complex image data^10–15^. CNNs can extract features from abstracted layer of filters and are used to analyze dental images such as X-rays, MRIs, and CT scans with high accuracy^9,16,17^, demonstrating promising application prospects in identifying dental caries^11,18^, vertical root fractures^19^, apical lesions^20,21^, periodontal bone loss^22^, types of implants in patients’ alveolar bone^23^, as well as the root and canal variation^13–15^. The aim of this study is to develop CNN-based deep learning models for the classification of two- and three-rooted MFMs on panoramic radiographs and to assess their diagnostic accuracy, with CBCT images serving as the gold standard (Fig. 1 shows the flowchart of the study).

## Results

### Diagnosis of three-rooted MFMs based on CBCT examination

**Fig. 2:**
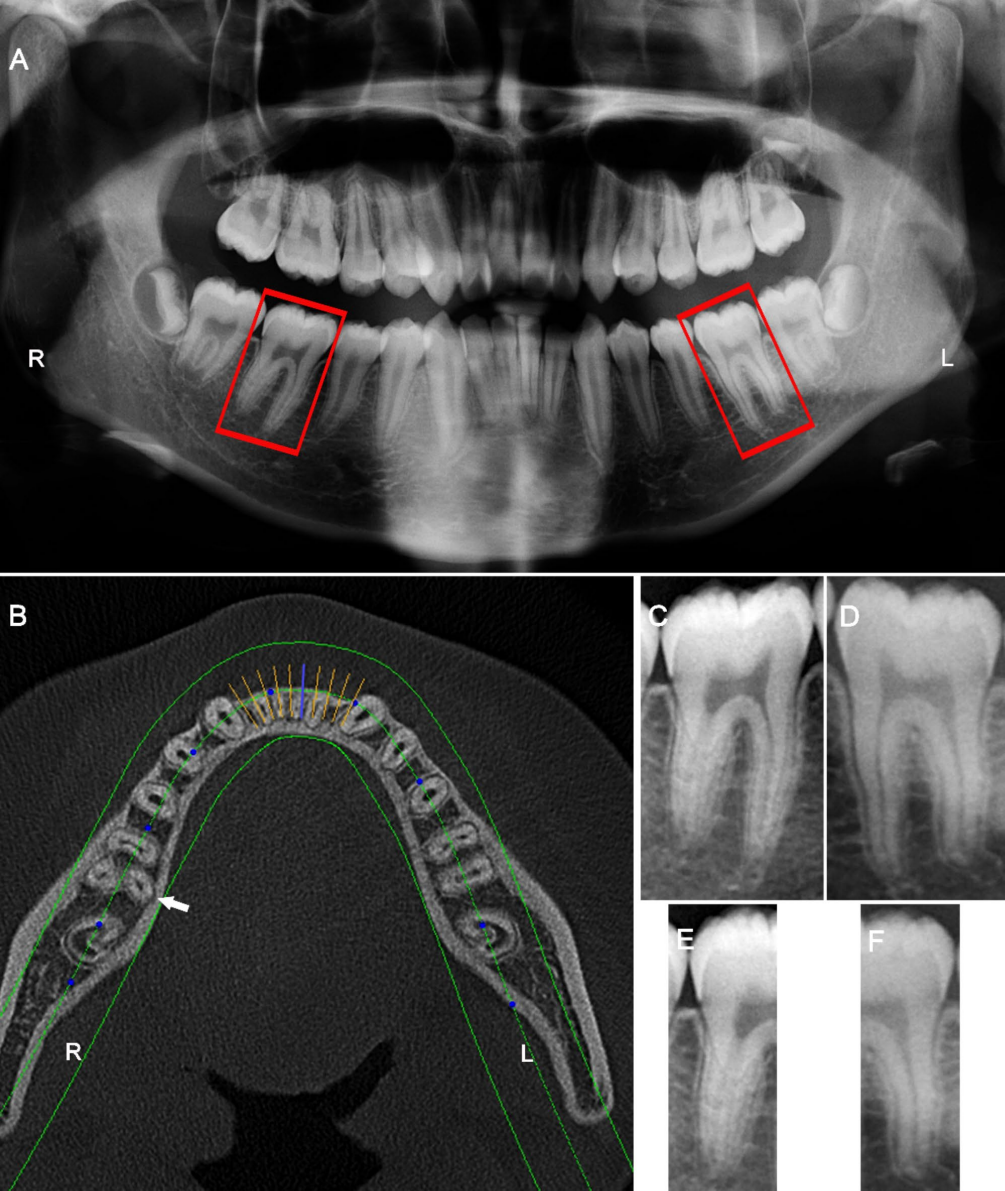
Preparation of image patches of mandibular first molars (MFMs) (The CBCT and panoramic images were derived from a same patient). (A) A panoramic radiograph; (B) an axial CBCT cross-section shows a three-rooted MFM (white arrow) on the right side and a two-rooted MFM on the left side (the gold standard for labeling image patches); (C and D) image patches of the right and left MFMs were cropped from the panoramic radiograph; (E and F) panoramic image patches of the distal halves of bilateral MFMs.

 The CBCT examination (depicted in Fig. 2B), serving as the gold standard, revealed that among a total of 1444 MFMs, 367 teeth (25.4%) were three-rooted and 1077 teeth (74.6%) were two-rooted. For three-rooted MFMs, the majority of mesial roots have two canals, while the distobuccal (DB) and DL roots consistently have a single canal, without exception.

### **Diagnostic performance of the deep learning system**

**Fig. 3:**
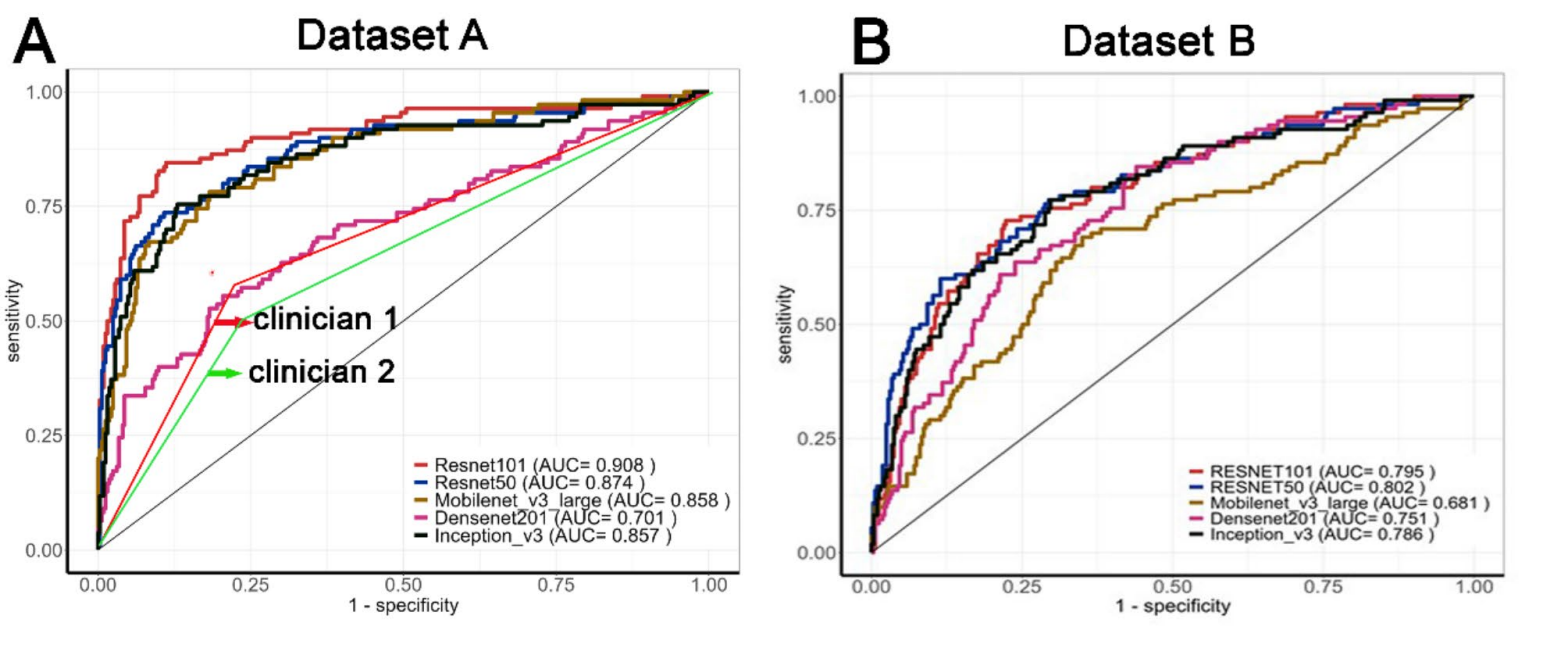
Receiver operating characteristic (ROC) curve analysis of five CNN models and two clinicians in classification of image patches of mandibular first molars (MFMs). (A) The CNN models were trained and tested with Dataset A (the image patches fully display the mesial and distal roots). (B) The CNN models were trained and tested with Dataset B (the image patches contain only the distal halves of MFMs).

The performance of the deep learning system in the diagnosis of three-rooted MFMs is shown in Table 1; Fig. 3. The overall performance of ResNet-101 was the best among the five CNNs. On Dataset A, ResNet-101 demonstrated the highest AUC value of 0.907, with an accuracy of 87.5%, sensitivity of 83.6%, and specificity of 88.9%. The Delong test indicated that the AUC is significantly (all *P* < 0.01) higher than that of all other networks. DenseNet-201, however, showed the lowest diagnostic performance among the five models (all *P* < 0.01), with an accuracy of 73.2% and an AUC value of 0.701. The performance of the two clinicians was even poorer, with AUC values of only 0.680 and 0.632, and accuracies as low as 73.0% and 70.0%, respectively.

**Table 1:** Diagnostic performance of five CNN models and two clinicians

CNN models	Acc	AUC	Asymptotic 95% confidence interval		Se	Sp	PPV	NPV	F1
			Lower bound	Upper bound			(Precision)		
Dataset A									
ResNet-101	0.875	0.907	0.869	0.945	0.836	0.889	0.719	0.941	0.773
ResNet-50	0.850	0.875	0.833	0.918	0.727	0.892	0.696	0.906	0.711
DenseNet-201	0.732	0.701	0.640	0.762	0.545	0.796	0.476	0.837	0.333
Inception-v3	0.838	0.862	0.818	0.905	0.745	0.87	0.661	0.909	0.701
Mobilenet-v3	0.806	0.857	0.813	0.900	0.773	0.817	0.590	0.913	0.669
Clinician 1	0.730	0.680	0.634	0.724	0.574	0.786	0.493	0.836	0.530
Clinician 2	0.700	0.632	0.584	0.677	0.487	0.777	0.441	0.807	0.462
Dataset B (distal halves of MFMs)									
ResNet-101	0.762	0.795	0.745	0.844	0.718	0.777	0.523	0.718	0.605
ResNet-50	0.810	0.800	0.751	0.851	0.582	0.885	0.630	0.860	0.610
DenseNet-201	0.630	0.750	0.700	0.802	0.827	0.560	0.390	0.910	0.530
Inception-v3	0.720	0.790	0.731	0.835	0.755	0.703	0.460	0.890	0.570
MobileNet-v3	0.660	0.680	0.622	0.741	0.673	0.656	0.400	0.860	0.500

Acc, accuracy; AUC, area under curve; Se, sensitivity; Sp, specificity; NPV, negative predictive value; PPV (precision), positive predictive value.

To assess whether the anatomy of the mesial aspect of MFMs contributes to image classification predictions by the deep learning system, we applied the five CNN models to Dataset B, which contains image patches restricted to the distal half of MFMs (Fig. 2E, F). Overall, the CNNs demonstrated notably reduced performance compared to Dataset A, with AUC values ranging from 0.680 to 0.800 and accuracy between 63.0% and 81.0% (Table 1).

### Explainability using Gradient-weighted Class Activation Mapping (Grad-CAM)

**Fig. 4:**
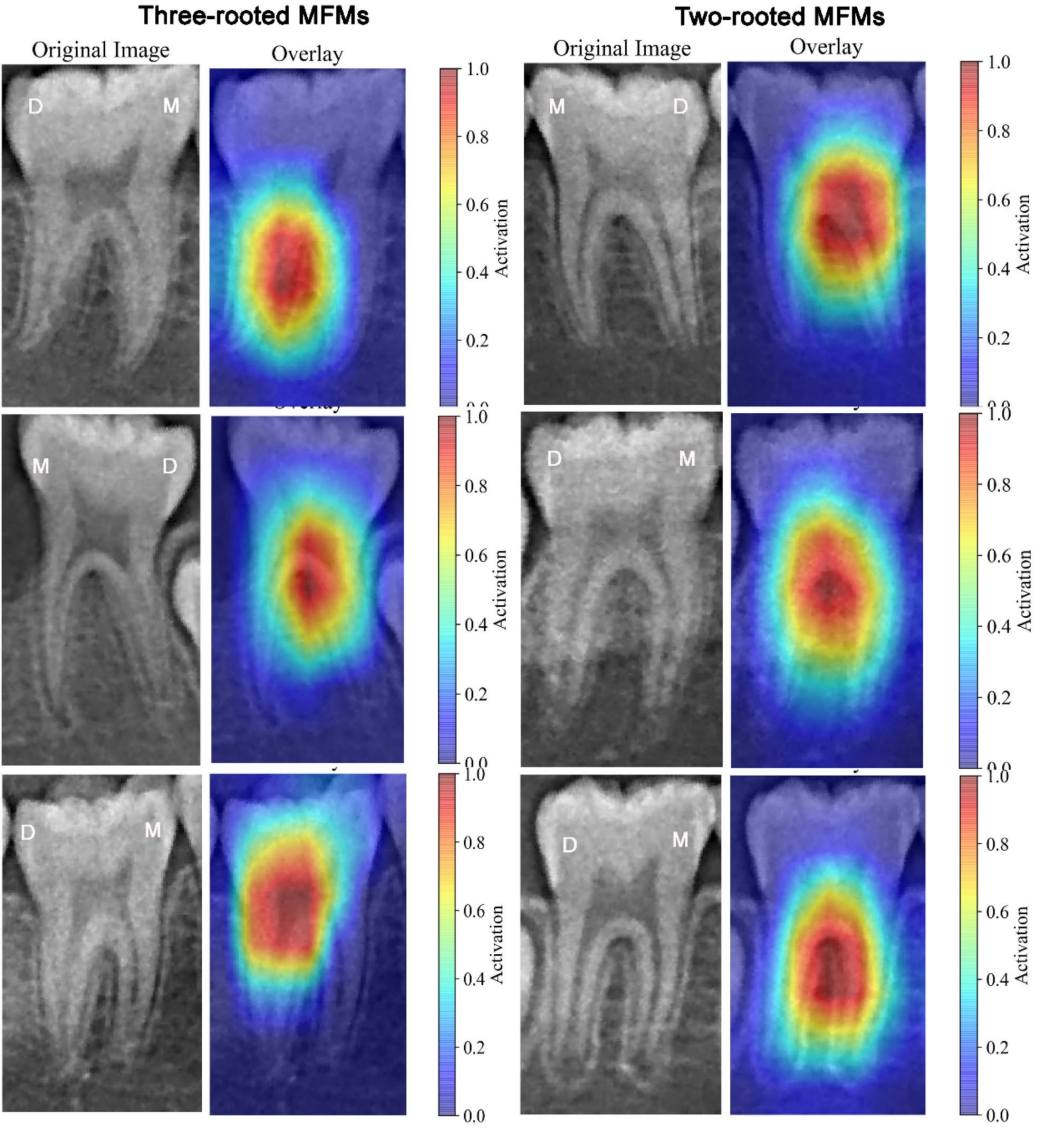
Representative activation maps of some correctly classified panoramic images (using ResNet-101). Class Activation Maps (CAMs) were drawn by applying Grad-CAM, and the heatmaps are focusing on the furcation area of MFMs, especially near the coronal two thirds of the distal root, which is the possible location of the DL root or the distal root furcation. The red areas indicate greater weighting.

 Grad-CAM analysis presented in Fig. 4 shows the key features selected for the classification task. Most CAMs highlighted the root furcation area, particularly focusing on the coronal two-thirds of the distal root, which represents the possible location of the additional DL root or the distal root furcation.

## Discussion

Recently, the deep learning system has been applied to various medical fields, and it shows multiple advantages when applied in dentistry^9,16^. In endodontics, predicting the root and canal variation through radiographs prior to root canal therapy is helpful in the treatment process. The prevalence of three-rooted MFMs is notably high among East Asians^4–7^, and in this report, the frequency was 24.6% (367/1444 teeth) in a Chinese population. However, it has been reported that a substantial percentage of this root trait cannot be discerned on preoperative radiographs^5^. In this study, a CNN-based deep learning system was used to predict the presence of three-rooted MFMs by analyzing panoramic radiographs. Unlike traditional methods, CNNs do not require manual feature extraction. They automatically learn to detect important features from raw data, which enhances the efficiency and effectiveness of the diagnostic process^24^. Moreover, CNNs are robust to variations in image quality and can process large volumes of images quickly and consistently, which is crucial in a clinical setting where time and accuracy are paramount. This scalability facilitates expedited diagnosis and treatment decisions^16,24^.

In the current study, five CNN architectures were employed and evaluated for their diagnostic performance on DL roots using panoramic images. As shown in Table 1, ResNet-101 demonstrated the best diagnostic performance (AUC = 0.907, accuracy = 87.5%), indicating a high prediction accuracy for DL roots. However, both senior and novice clinicians achieved lower diagnostic performance, with an AUC of 0.680 and 0.632, respectively (*P* > 0.05), and an accuracy of 73.0% and 70.0%, respectively. Professional experience did not appear to significantly contribute to better results. This finding also indicates that ResNet-101 can be a promising tool to assist clinicians in the detection of three-rooted MFMs on panoramic radiographs, thereby mitigating the necessity for CBCT examination, which confirms the principle of ALARA or ALADA. ResNet-101 is part of the ResNet (Residual Neural Network) family, known for its use of residual connections to address the vanishing gradient problem. ResNet-101 has 101 layers and is designed for very deep learning, making it suitable for complex image recognition tasks. ResNet-50, a smaller and faster version with 50 layers, offers a good trade-off between depth and computational efficiency and is often used in applications requiring less computational power^25^. In this study, ResNet-50 achieved superior performance (AUC = 0.875) compared to most other models, though it was only slightly inferior to ResNet-101. Among the five networks, DenseNet-201 showed the lowest diagnostic performance, with an AUC value of only 0.701, which is close to the value obtained by the senior endodontist (AUC = 0.680), indicating that DenseNet-201 can’t significantly improve diagnostic accuracy. DenseNet connects each layer to every other layer in a feed-forward fashion, encouraging feature reuse and significantly reducing the number of parameters. DenseNet-201, with 201 layers, is particularly effective in mitigating the vanishing gradient problem and achieving high performance with fewer parameters^26^. However, our data indicate that it does not meet the requirements of the current task. Our findings demonstrated that ResNet-50, Inception-v3 and MobileNet-v3 could achieve a moderate diagnostic potential with AUC values ranging from 0.857 to 0.875, which is significantly greater than those of the clinicians. MobileNet-v3 is optimized for mobile and edge devices. It offers a balance between latency, accuracy, and model size, making it ideal for real-time applications on resource-constrained devices^27^. In contrast, Inception-v3 is often used in image classification tasks where high accuracy is crucial. Its complex architecture uses various types of convolutions and pooling operations within the same layer. It includes auxiliary classifiers to help with training deeper networks and achieves high accuracy with relatively fewer parameters^28^. Recently, a similar experiment was performed on a Japanese population by Hiraiwa et al.^12^, who employed two other CNN-models (AlexNet and GoogleNet) for the classification of two- and three-rooted MFMs on panoramic radiographs. They reported that AlexNet demonstrated an accuracy of 87.4%, sensitivity of 77.3%, specificity of 97.1%, and an AUC value of 0.87, while GoogleNet achieved an accuracy of 85.3%, sensitivity of 74.2%, specificity of 95.9%, and an AUC value of 0.85. These models exhibit diagnostic potential slightly lower than that of ResNet-101 in our study and are comparable to the performance of ResNet-50, Inception-v3, and MobileNet-v3. Jeon et al.^15^ applied another CNN model (Xception architecture) for predicting C-shaped canals in mandibular second molars on panoramic radiographs, achieving a higher accuracy of 95.1% (AUC = 0.982), compared to 87.3% (AUC = 0.872) for the radiologist and 88.5% (AUC = 0.885) for the endodontist. Taken together, each of these models has its strengths, and the choice of model often depends on the specific requirements of the task, such as the need for accuracy, computational resources, and the context in which the model will be deployed.

It has been well known that the inner workings and decision-making processes of CNNs remain opaque and difficult to understand, hence being described as a “black box”^29^. In this study, Grad-CAM was used to make the CNN-based model more transparent through visual explanations. It highlights the regions in an input image that are important for the prediction of a specific class. It is achieved by computing the gradient of the class score with respect to the feature maps of a convolutional layer. The gradients indicate which pixels in the feature maps are most relevant for the decision. The gradients are then pooled to obtain a weight for each feature map. These weights indicate the importance of each feature map for the class under consideration. The weighted feature maps are then combined to produce a coarse localization map (heatmap) of the important regions in the image, which is overlaid on the original image. This visualization shows the areas in the image that the model is focusing on when making a prediction, helping to understand why the model made a certain decision^30^. In this study, as shown in Fig. 4, the heatmaps highlight the root furcation area, especially near the coronal two-thirds of the distal root, which is the possible location of the DL root or distal root furcation. Previous scholars have demonstrated that the DL root is frequently smaller in size and overlapped by the DB root on the conventional radiograph (where both roots possess a single canal) and careful reading of angled radiographs can enhance the detection rate^31,32^. The criteria for the indication of an additional DL root were justified by the crossing of the translucent boundaries delineating the root canal space and the periodontal ligaments in MFMs^33,34^. However, we found that a significant proportion of the cases of DL roots were overlooked by the clinicians, with the sensitivity rates of only 57.4% and 48.7%, respectively, which are substantially lower than the 83.6% achieved by ResNet-101. By examining the heatmaps, we have found that in most cases, the model focuses on the relevant parts of the image. The insights gained from Grad-CAM can be utilized to improve the model architecture and training processes^29,30^.

Since the DL root consistently resides posterior to the DB root, often fully or partially obscured by the latter, the clinicians’ attention is always on the distal portion of MFMs when reading a conventional two-dimensional radiograph, especially focusing on the radiographic details behind and around the anticipated DB root. To disclose whether and how much the mesial portion of MFMs influences the diagnostic accuracy of CNNs, we applied the deep leaning models on Dataset B (image patches including only the distal half of MFMs). Surprisingly, we encountered a notable decline in the diagnostic performance of the CNNs, with AUC values falling below 0.800 and accuracy ranging from 63.0 to 81.0%. This finding suggests that the mesial half of MFMs contributes to the classification of two- and three-rooted MFMs, and encompasses critical features that can be recognized by CNN networks, but not by the eyes of clinicians. The heatmaps (Fig. 4) confirm that the highlighted regions frequently involve the mesial portion of the teeth (particularly the furcation area on the mesial side). This finding also partially explains why CNNs outperformed clinicians in detection of this root variation.

This study has several limitations. First, the preparation of image patches relied on manual segmentation, which is a time-consuming process and may hinder its clinical application. AI-assisted image segmentation has been widely employed in other fields^15,23^. For example, the You Only Look Once (YOLO) algorithm can efficiently segment regions of interest (ROIs) within medical images, encompassing implants, tumors, organs, and blood vessels, while also rapidly and accurately classifying these ROIs^23^. This capability is crucial for disease diagnosis, treatment planning, and prognosis assessment. Recently, some scholars have constructed models that separate detection and classification into two steps^23^, and automating this process appears to be the most optimal solution currently available. Second, labeling each image (for supervised learning) was also a labor-intensive job, and can limit the available amount of data for training and reduce the models’ accuracy. Unsupervised or active learning may help to overcome these limitations^35^. Third, although Grad-CAM can visualize class activation and help in the evaluation of features, it relies on gradient information, which can be inaccurate or noisy, potentially misleading the true importance of features. Moreover, the results of Grad-CAM can vary with different model architectures, leading to inconsistent feature evaluations^29,36^. Therefore, it should be used with caution and supplemented with other methods for a comprehensive assessment. Finally, in the current study, all the subjects were Chinese, and since root variation is influenced by ethnicity, further multi-centered studies are essential, including more subjects from other geographical regions and racial backgrounds.

## Conclusion

In conclusion, the CNN-based deep-learning system demonstrated high accuracy in the detection of three-rooted MFMs on panoramic radiographs.

## Materials and methods

### Data acquisition

Imaging data from 788 subjects, who underwent panoramic radiography together with dental CBCT examination (Fig. 2) between July 2020 and July 2024, were randomly selected from our hospital’s image database. All examinations were performed for diagnostic reasons before or during dental treatment and were not solely acquired for the purpose of this study, thereby avoiding unnecessary radiation exposure. The inclusion criteria were as follows: (1) known gender and age; (2) each subject had bilateral MFMs. The exclusion criteria were as follows: (1) obvious artifacts on CBCT or panoramic images; (2) MFMs with root canal fillings, posts, or crowns. A total of 730 patients (353 males and 377 females) with 1444 MFMs were selected for further experiments based on the inclusion and exclusion criteria. The mean age of the enrolled subjects was 24.6 ± 10.4 years, ranging from 10 to 66 years.

CBCT examination was performed using a 3D eXam i CBCT machine (KaVo, Germany). The imaging protocol was as follows: FOV = 14 × 8.5 cm; tube peak potential = 120 kVp; tube current = 30.89 mA; acquisition time = 23 s; voxel size = 0.25 mm. KaVo eXam Vision software (KaVo, Germany) was used to analyze the images, and generate simulated panoramic images. Panoramic images were obtained using a Planmeca Promax 3D Classic system (Planmeca Oy, Helsinki, Finland) with the standard parameters, including a tube voltage of 66 kV, tube current of 8 mA, and acquisition time of 15.8 s.

### Classification of MFMs according to CBCT examination

The CBCT images served as the gold standard for diagnosing the root number of MFMs. At the interface of the Kavo eXam Vision software, one observer (*Y. G.*) accurately determined the presence or absence of an additional DL root in MFMs by meticulously examining a series of axial images, progressing from the pulp chamber to the apex (Fig. 2B). Based on this examination, the teeth were classified into two categories: two-rooted and three-rooted MFMs. Cohen’s kappa test was used to evaluate the intra-observer errors. In a piolet study, the observer evaluated the root number for 50 MFMs twice, with a 14-day interval. The intra-observer error kappa value was 1.0.

### Preparation of image patches of MFMs from panoramic images and annotation

On panoramic images, the right and left MFMs were segmented using rectangles in Photoshop CS3 software (Adobe Systems Incorporated, San Jose, USA). The rectangular image patches were required to fully display the mesial and distal roots of the MFM (Fig. 2A, C and D).

The image patches were classified and labeled into two categories: two-rooted and three-rooted MFMs, based on the CBCT examination. Subsequently, they were de-identified and anonymized before further analysis. The final image dataset (Dataset A) comprised 367 three-rooted MFMs and 1077 two-rooted MFMs. Dataset A was subsequently replicated and backed up. In the replicated dataset, termed Dataset B, each image patch of the MFM was further cropped into a rectangle and the ROI contained only the distal half of the MFM (Fig. 2E and F).

### Architecture of the CNN-based deep learning system

The network architecture was designed using the Pytorch machine learning framework (version 1.13.1), and the training was performed on a GPU. The modelling was implemented on an Intel Core i9-13900KF CPU (Intel, USA) and an NVIDIA GeForce RTX 4090 GPU (NVIDIA, USA) with 24 GB GDDR6X memory. The framework utilized was CUDA and CuDNN, and the programming language used was Python.

To classify two- and three-rooted MSMs, five CNN models (ResNet-101 and −50 ^25^, DenseNet-201^26^, MobileNet-v3^27^ and Inception-v3^28^) were employed. These CNN algorithms were pre-trained on the 2012 ILSVRC ImageNet database^37^.

A Python code snippet was executed to randomly allocate the panoramic image patches into training and validation datasets in a ratio of 7:3, ensuring that training patches did not overlap with the validation dataset. In Dataset A/B, 110 images of three-rooted MFMs and 323 images of two-rooted MFMs were used for evaluation, and the remaining 257 images of three-rooted MFMs and 754 images of two-rooted MFMs were used for training (Fig. 1).

The image datasets of MFMs were resized to a resolution of 224 × 224 pixels using the Transform sub-library of the Torchvision library (version 0.14.1) and normalized to a fixed range (0, 1). The SGD algorithm was employed as the optimizer with an initial learning rate of 0.1, a batch size of 64, and batch normalization. Each model was trained for 50 epochs per k-fold cross-validation, for a total of 500 epochs, with random splitting of the training and validation sets. The output layer of a CNN model for a binary classification problem consists of a single neuron with a sigmoid activation function. The sigmoid function produces a value between 0 and 1, representing the probability that the image belongs to the positive class. A default threshold of 0.5 is commonly used: if the sigmoid output is greater than or equal to 0.5, the image is predicted to belong to the positive class; otherwise, it is predicted to belong to the negative class. In the current study, by analyzing the receiver operating characteristic (ROC) curve and the area under the curve (AUC) score, a more suitable threshold was selected to balance precision and recall.

To better understand the effect of the network, the classifier networks were further assessed using Grad-CAM^28^ to provide a visual localization of class-discriminative regions.

### Comparisons between CNN models and clinicians

To compare the diagnostic performance of the deep learning system with the clinical diagnosis, a senior endodontist (Clinician 1, *Y. G.*, with over 20 years of experience) and a novice general dentist (Clinician 2, *W. Z.*, with two years of experience) independently predicted the presence of three-rooted MFMs on panoramic radiographs, blinded to the true labels. In the piolet study, Cohen’s kappa test was employed to evaluate the inter-observer agreement; following training on several panoramic images, both observers evaluated the root number for 50 MFMs, yielding an inter-observer kappa value of 0.626. Subsequently, the same testing dataset utilized for the ResNet-101 model was employed, consisting of 433 image patches selected from Dataset A. The two clinicians then conducted actual observations to classify the MFMs as either three-rooted (scored as 1, positive case) or two-rooted (scored as 0, negative case).

### Statistical analysis

ROC curve analyses were performed to evaluate the classification performance, and DeLong test was used to compare the AUC values. *P* < 0.05 was considered statistically significant. The diagnostic performances of the networks and clinicians were evaluated with following standard metrics, including accuracy, sensitivity, specificity, precision, and F1 score.

accuracy = (TP + TN)/(TP + TN + FP + FN)

sensitivity (recall) = TP/(TP + FN)

specificity = TN/(TN + FP)

precision = TP/(TP + FP).

​ F1 = 2 × (Precision × Recall) / (Precision + Recall)​

(TP = True Positive; FP = False Positive; TN = True Negative; FN = False Negative).

## Data Availability

The data that support the findings of this study are available upon request from the corresponding author.
